# Current status of human biomonitoring of beauvericin and enniatins

**DOI:** 10.1007/s00204-026-04527-4

**Published:** 2026-09-01

**Authors:** Gsela H. Degen, Anamarija Romac

**Affiliations:** 1 Leibniz Research Centre for Working Environment and Human Factors (IfADo), Ardeystrasse 67, 44139 Dortmund, Germany; 2 Zagreb, Croatia

**Keywords:** Beauvericin, Biomonitoring, Enniatins, Food contaminants

## Abstract

Beauvericin (BEA) and enniatins (ENNs) are *Fusarium* mycotoxins found in cereals and cereal-based foods as reported in several studies. But information on human internal exposure remains scattered. Human biomonitoring (HBM) enables the investigation of exposure to these nonregulated contaminants. This review summarizes data from studies published since 2014 on occurrence of BEA and ENNs in human urine, blood, breast milk, tissues and hair. Most studies from Europe, Africa, Asia and North America applied state-of-art methodologies for biomarker analysis. ENN B was the most frequently detected compound and was found in a high proportion of blood samples across different populations. Although BEA was reported less frequently, measurable levels were found in plasma, breast milk, tissues and, more recently, in urine. Both BEA and ENNs were found in human milk, indicating transfer from mother to infant, but generally at low concentrations. Data for human tissues suggest differences in distribution, with BEA occurring mainly in adipose tissue and ENN B reaching the highest concentrations in liver. Some studies reported phase-I metabolites of ENNs in urine, demonstrating that biotransformation contributes to their elimination in humans. Available HBM data indicate widespread dietary exposure to BEA and ENNs in several human populations. Concentrations measured in blood and tissues are much lower than those associated with their cytotoxic effects in vitro. Uncertainties remain regarding human toxicokinetics and the toxicological relevance of their metabolites. Further studies addressing these gaps will improve interpretation of biomonitoring data and support the human health risk assessment of these emerging mycotoxins.

## Introduction

**Fig. 1:**
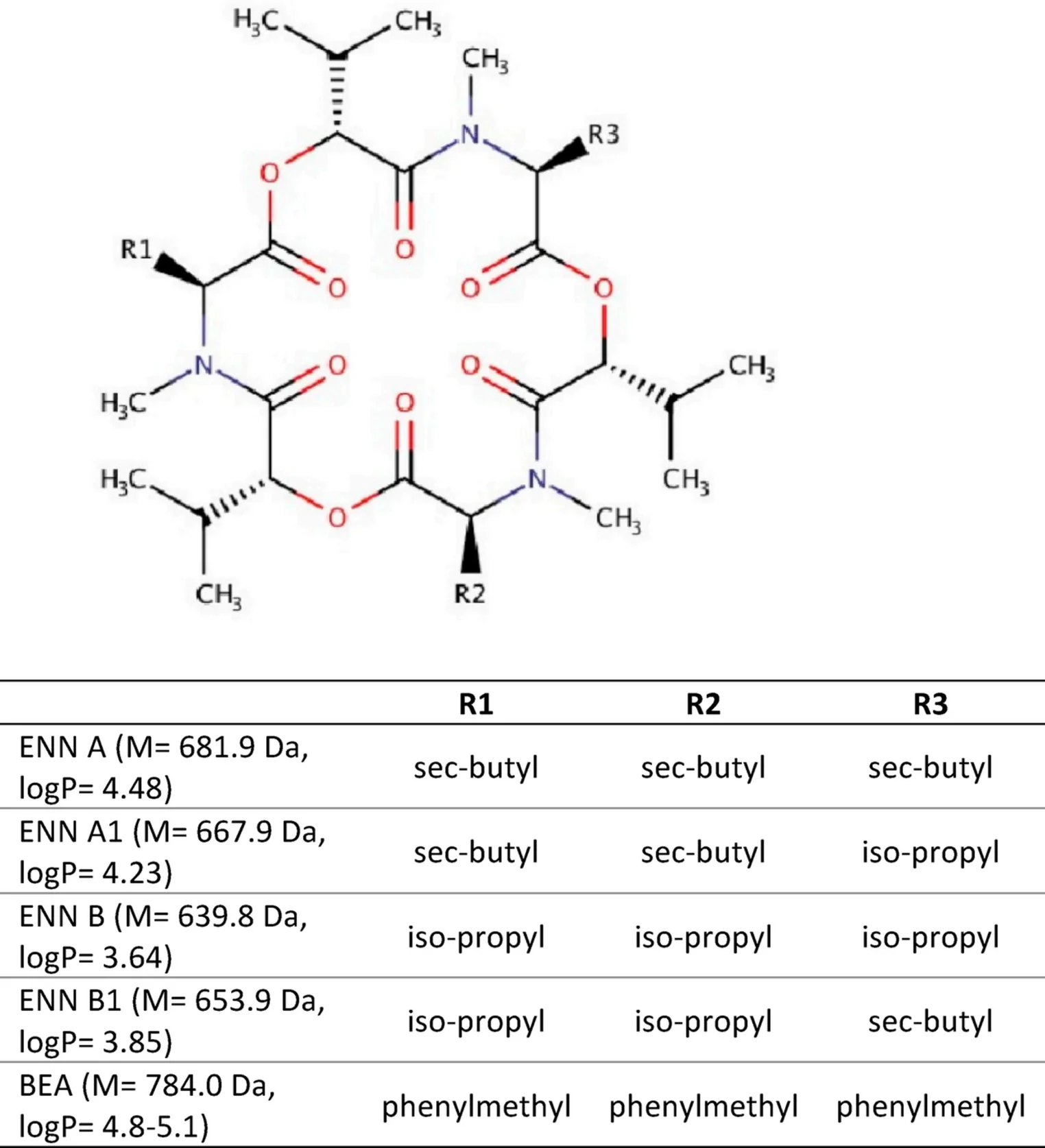
Chemical structures of beauvericin and enniatins

Beauvericin (BEA) and enniatins (ENNs), produced mainly by various *Fusarium* species, have gained interest in the last two decades as so-called ‘emerging’ mycotoxins, and are now recognized as frequent contaminants of food and feed in many regions of the world (Annunziata et al. 2025; Fraeyman et al. 2017; Jestoi 2008; Kolawole et al. 2024). The structurally related fungal products BEA, enniatin B (ENN B), enniatin B_1_ (ENN B1), enniatin A (ENN A), enniatin A_1_ (ENN A1) are cyclohexadepsipeptides with relatively high lipophilicity and an amphiphilic ring structure (Fig. 1).

These mycotoxins show antibacterial, antimycotic and insecticidal activity, effects related to their ionophoric properties (EFSA CONTAM Panel 2014). Moreover, BEA and ENNs exert cytotoxicity in vitro at low µM concentrations in different mammalian cell types (data reviewed in Bertero et al. 2020; Behr et al. 2025). Cytotoxicity could lead to local and/or systemic adverse effects in cases of very high dietary mycotoxin exposures. On the other hand, concerns about a genotoxic potential of BEA can be dismissed based on new and existing data and evaluations which indicate a lack of such activity for this contaminant (EFSA CONTAM Panel 2024). The genotoxic potential of ENN B was investigated previously in various in vitro tests with negative results (EFSA CONTAM Panel 2014). Results of in vitro genotoxicity tests with other ENNs and an in vivo study with oral ENN B administration have been summarized recently (Behr et al. 2025), with negative outcomes in vivo for multiple endpoints*,* yet some inconsistent findings in vitro for structurally closely related ENNs.

EFSA’s CONTAM Panel has estimated the human dietary exposure to BEA and the sum of major ENNs, and concluded that the most important contributors are grains and grain-based products followed by dried fruits (EFSA CONTAM Panel 2014). The highest acute human exposure estimates for BEA (and the sum of ENNs were far (1,000,000 and 350,000 fold, respectively) lower than LD_50_ values reported for acute toxicity in mice (100 mg/kg b.w. for BEA and 350 mg/kg b.w. for fusafungine; a mixture of enniatins). Yet, given the lack of relevant in vivo toxicity data in repeated dose animal studies, a full risk assessment was not possible in 2014, and it still presents a challenge due to major data gaps in the hazard characterization of these mycotoxins and their toxicokinetics, especially in humans.

Additional studies for an in-depth hazard characterization both a series of in vitro tests and a 90-day feeding study with ENN B1 are conducted in an ongoing research project within the Partnership for the Assessment of Risk from Chemicals (PARC), yet publication of the results is not expected before end of 2026. A recent study from PARC investigated in vitro hepatic clearance of ENN B1 comparing human and rat cell models (Mahdjoub et al. 2026). Yet, a toxicokinetic in vivo study in humans has not published so far.

Toxicokinetic information on oral absorption, distribution, metabolism and excretion (ADME) is needed for a risk assessment, as these processes influence the bioavailability (*internal* dose) of an ingested contaminant. In animal studies, reviewed recently by Behr et al. (2025), a wide range of values is observed for the bioavailability of BEA and ENNs in different species. Systemic exposure in humans is thus difficult to predict, also considering differences in the mycotoxins ‘ biotransformation rates by liver microsomes from several mammalian species including human (ENNs: Faeste et al. 2011; Ivanova et al. 2011, 2017, 2019; Bhateria et al. 2022; BEA: Yuan et al. 2022; Pandey et al. 2025a).

BEA and ENNs are not monitored routinely because of an absence of regulatory limits for these contaminants, although their occurrence is often detected in various foods, including cereal-based infant foods (Annunziata et al. 2025; de Sá et al. 2025). This raises the question of potential risks from dietary exposure to these *Fusarium* toxins and how this may be reflected in human biological samples.

Human biomonitoring (HBM) by suitable analytical methods allows to determine the presence of parent compounds and metabolites in biological matrices (urine, blood and breast milk). This approach is now widely applied in the field of mycotoxin research as it captures all sources of exposure and reflects also the influence of toxicokinetics (Cramer et al. 2026). Since analytical methods for a sensitive detection of BEA and ENNs in human samples have been published first in 2014 and thereafter, such data were not available for a first risk assessment conducted by EFSA for these emerging mycotoxins (EFSA 2014).

This review compiles data from HBM studies in different countries where the occurrence of BEA and ENNs has been analyzed in urine, blood (plasma or serum), and breast milk samples, as well as data on their presence in several human tissues. The information available so far provides valuable insights into the fate of these mycotoxins in the human organism; internal concentrations of BEA and ENNs resulting from dietary exposure are discussed in the context of existing information on cytotoxicity and on internal levels in animals; further research needs will be also addressed.

## Methods

Information on presence of BEA and ENNs in human samples was gathered in multimodal ways, starting with early papers on their analysis in blood, in breast milk or urine (Osteresch et al. 2017; Braun et al. 2018; Gerding et al. 2014; Serrano et al. 2015), and tracing publications which cited them. In addition, more recent publications in the PubMed, Scopus and Web of Science databases on mycotoxin biomonitoring were examined whether the applied multi-mycotoxin methods included an analysis of BEA and/or ENNs. The literature search served as the basis for a narrative review and covers publications (in English) up to June 2026.

## Results

State-of-the-art methods for determining the occurrence of mycotoxins in biosamples rely on liquid chromatography separation coupled with mass spectrometric analysis after sample preparation and clean-up (Cramer et al. 2026; Hernandez-Mesa et al. 2026). Protocols used for an enrichment of analytes, *e.g.* liquid/liquid or solid phase extraction, vary with sample type and in sensitivity for detecting biomarkers^1^ of exposure in urine, blood plasma/serum or in breast milk samples. Presence of BEA and ENNs in these biological matrices has been mainly investigated by multi-mycotoxin methods. Some studies have applied targeted methods for an analysis of parent compounds and/or metabolites in human samples (Serrano et al. 2015; Rodríguez-Carrasco et al. 2018, 2020; Castell et al. 2024).

The outcome of HBM studies conducted so far is given in Tables 1, 2, 3, and 4 along with brief information on population groups, methods applied and achieved detection levels. Results in the different matrices analyzed are summarized first for BEA, then for ENNs.

**Table 1:** Analysis of BEA and ENNs in spot URINE Samples

Authors/Country/Study Group & Sample numbers	Analyte	% (n) detected	Mean/Median (Range) µg/L or creatinine adjusted	LOD/LOQ µg/L	Comments
Gerding et al. (2014); Germany, n = 100 adults (Münster)	ENN B	20%	> LOD but < LOQ	0.005/0.0125	Multi-mycotoxin method (BEA not included)
Gerding et al. (2015); Germany, n = 50 adults (Dortmund) Haiti, n = 142 volunteers Bangladesh, n = 95 adults	ENN B	14%	0.012 ± 0.002 µg/L (0.017 µg/g crea)	0.00125/0.005	Multi-mycotoxin method (BEA not included)
	ENN B	4%	0.036 ± 0.020 µg/L (0.029 µg/g crea)	0.00125/0.005	
	ENN B	2%	0.019 ± 0.000 µg/L (0.015 µg/g crea)	0.00125/0.005	
Serrano et al. (2015); Italy; n = 10 adults	BEA	n.d	n.d	0.010/0.03	Method development for BEA and ENNs in urine (and plasma)
	ENN A, A1	n.d	n.d	0.010/0.03	
	ENN B, B1	1 of 10	Traces of EnnB1	0.005/0.01	
Escrivá et al. (2017); Spain; n = 10 adults	BEA	0/10	n.d	0.3/1.0	Method development for optimized extraction of multiple mycotoxins
	ENN B	4/10; 4 <LOQ	0.1–0.54 µg/L	0.05/0.1	
	ENN B1	2/10; 4 <LOQ	–0.34 µg/L	0.05/0.1	
	ENN A	1/10 at <LOQ	n.d	0.2/0.5	
Rodríguez-Carrasco et al. (2018); Italy; n = 300 adults	ENN B	83.7%	0.016 (0.006–0.391) µg/L	0.0005/0.001	ENN B & ENN B metabolites: mono-oxygenated forms N-demethylated forms dioxygenated forms
	M1–M5	87.7%	0.033 (0.005–0.107)		
	M6–M7	96.3%	0.020 (0.002–0.102)		
	M8–M12	6.7%	0.051 (0.001–0.552)		
Rodríguez-Carrasco et al. (2020); Italy; n = 300 adults	ENN B1	94.3%	0.069 (0.007–0.429) µg/L	0.0005/0.001	ENN B1 & ENN B1 metabolites: demethylated & hydroxylated hydroxylated forms carbonylated forms carboxylated forms
	M1	5.3%	0.035 (0.007–0.177)		
	M2–M5	78.0%	0.069 (0.006–0.233)		
	M6–M8	66.0%	0.196 (0.012–1.763)		
	M9–M11	26.3%	0.066 (0.008–0.656)		
Liu et al. (2020); China; n = 60 adults (26 m, 34 f; age 4–70 y)	BEA	0%	n.d	0.0002/0.0006	Multi-mycotoxin method by UHPLC-MS/MS of hydrolysed urines after SPE clean-up
	ENN B	40%	0.0001 (> LOD–0.0014) µg/L	0.0001/0.0002	
Warensjö-Lemming et al. (2020); Sweden; n = 1096 adolescents	ENN B	<LOD	n.d	0.01/0.03	Multi-mycotoxin method
Arroyo-Manzanares et al. (2020); Spain; n = 10	BEA,	< LOD	n.d	0.01/0.04	SPE with magnetic multiwalled carbon nanotubes prior to UHPLC-HRMS
	ENN A, A1	< LOD		0.03/0.01	
	ENN B, B1	< LOD		0.01/0.04	
Penczynski et al. (2022); Germany; n = 36 vegans and n = 36 omnivores	BEA	<LOD	n.d	0.1/0.3	Multi-mycotoxin method; ENN B **<** LOD in 24 h urines, but present in Serum (see Table 2)
	ENN B	<LOD	n.d	0.01/0.03	
Dasi-Navarro et al. 2022; Spain; n = 100 women	BEA	0%	Below LOD	1.2/3.8	Multi-mycotoxin method (HPLC-Q-TOF-MS)
	ENN B	27%	3.5 ± 1.0 µg/g crea (0.5–2.5 µg/L)	0.1/0.3	
	ENN B1	5%	1.3 ± 0.4 µg/g crea (0.5–0.8 µg/L)	0.1/0.3	
Dasí-Navarro et al. (2024); Spain; n = 524 women	ENN B	26%	4.8 ± 4.8 (1.0–39.7) µg/g crea	Not reported	Multi-mycotoxin method (HPLC-Q-TOF-MS); (BEA not included)
	ENN B1	7%	2.1 ± 2.7 (0.5–14.4) µg/g crea		
	ENN A	2%	0.7 ± 0.6 (0.3–2.1) µg/g crea		
	ENN A1	<1%	2.0 ± 0.9 (1.1–2.8) µg/g crea		
Gallardo-Ramos et al. (2024); Spain; n = 20 donors of each 4 urines	ENN B	68.75%	4.36 (0.07–4.45) µg/L 2.4 µg/g crea	0.01/0.03	Multi-mycotoxin method (BEA not included)
Mihalache et al. (2026); Italy; n = 300 urine donors (57% women, 43% male)	BEA	96%	0.70 ± 0.57 (0.28–7.38) µg/L 0.61 ± 0.49 (0.07–5.09) µg/g crea	0.04/0.13 µg/L	Multi-mycotoxin method (LC–MS/MS) analysis after enzymatic hydrolysis of spot (morning) urines Volumetric concentrations listed in Supplement Table S3
	ENN B	90.6%	0.76 ± 0.40 (0.65–7.14) µg/L 0.76 ± 0.57 (0.14–12.02) µg/g crea	0.04/0.13 µg/L	
	ENN B1	94%	0.74 ± 0.29 (0.67–5.42) µg/L 0.73 ± 0.63 (0.14–9.13) µg/g crea	0.04/0.13 µg/L	
	ENN A	41.6%	0.71 ± 0.59 (0.52–4.82) µg/L 0.68 ± 0.48 (0.11–6.63) µg/g crea	0.03/0.10 µg/L	
	ENN A1	62%	0.73 ± 0.27 (0.65–3.68) µg/L 0.72 ± 0.65 (0.14–6.19) µg/g crea	0.04/0.13 µg/L

**Table 2:** Analysis of BEA and ENNs in Blood Samples (Serum/Plasma)

Authors/Country/ Study Group & Sample number	Analytes	% (n of) determined’	Mean/Median (Range) µg/L	LOD/LOQ µg/L	Comments
Osteresch et al. (2017); Germany; n = 50 adults	BEA	Not reported	Not reported	0.0014/0.05	Multimycotoxin analysis for dried serum spots
	ENN B	100%	0.033 (0.014–0.171)	0.0012/0.01	
Viegas et al. (2018); Portugal; n = 42 worker (waste sorting)	ENN B	100%	0.048 (0.014–0.176)	LOQ of 0.01	Method of Osteresch et al. (2017)
Warensjö-Lemming et al. (2020); Sweden; n = 1096 adolescents	ENN B	84.1% > LOQ	0.014 ± 0.012 *max. *0.113	0.0013/0.005	Multi-mycotoxin method for serum
Penczynski et al. (2022); Germany; n = 36 vegans and n = 36 omnivores	BEA	< LOD	n.d	0.014/0.05 0.0013/0.005	Multi-mycotoxin method for serum; ENN B was below LOD in 24-h urines of individuals (Table 1)
	ENN B in vegans	92% > LOQ	0.017 (0.001–0.104)		
	In omnivores	89 89% > LOQ	0.024 (0.001–0.096)		
Tesfamariam et al. (2022); Ethiopia; n = 579 pregnant women serum	ENN B	47% ≥ LOD	< LOD (< LOD–2.3)	Not reported (0.57/1.0)	Multi-mycotoxin method for serum by De Ruyck et al. 2020
Ning et al. (2024); China; n = 184 males	BEA	68.5% > LOD	0.117 (0.087–0.174)	0.005/n.r	Multi-mycotoxin method for plasma analysis
	ENN B	77.2% > LOD	0.006 (0.002–0.147)	0.001/n.r	
Yang et al. (2024); China; n = 192 males	BEA	> 60% pos	0.106 (0.003–1.121	0.005/0.01	Multi-mycotoxin method for plasma analysis
	ENN B	> 60% pos	0.003 (0.002–0.005)	0.001/0.002	
Krausová et al. (2024); USA; n = 50 pregnant females provided 200 serum samples	BEA	n.d	n.d	0.023/0.046	Multi-mycotoxin method for serum analysis (UPLC-MS/MS)
	ENN B	97%	0.02 (0.01–0.08)	0.003/0.006	
	ENN B1	12%	0.004 (0.003–0.007)	0.003/0.006	
Vermeire et al. (2025); Belgium; n = 52 adults and plasma samples	ENN B	125.9%	0.03 (0.01–0.029)	0.01/0.02	Multi-mycotoxin method for plasma analysis (UPLC-MS/MS)
Kyei et al. (2026); Bangladesh; n = 712 plasma samples (women & children)	BEA	5.9% ≥ LOD	Not reported	0.013/NR	Multi-mycotoxin method for plasma
	ENN B	9191.7% ≥ LOD	Not reported	0.001/NR	

*NR* = *not reported*

**Table 3:** **Analysis of BEA and ENNs in Breast Milk** Samples

Authors/Country/ Groups & samples	Analytes	% of detects	Mean/Median (Range) ng/L	LOD/LOQ ng/L	Comments
Braun et al. (2018); Nigeria; 22 women and n = 75 samples	BEA	56%	up to 19	LOD 6	Multimycotoxin method with limited sensitivity for some analytes
	ENN B	9%	up to 9	LOD 4	
Braun et al. (2020a); Austria; pooled BM sample	BEA	–	5.4	LOD 0.1	Method optimized; improved sensitivity for several analytes
	ENN B	–	3.7	LOD 0.7	
Braun et al. (2020b); Austria; (A): one mother n = 87 samples (longitudinal design) (B): 3 mothers on 5 days (n = 15 milk samples)	BEA	100%	1.2 (0.8–1.7)	LOQ 0.3	Study A: nursing up to 211 days; infant exposure estimated Study B: inter-day & interindividual variability on 5 consecutive days;
	ENN B	100%	2.3 (< LOQ–8.6)	LOQ 1.3	
	ENN B1	47%	1.9 (< LOQ–1.9)	LOQ 1.0	
	BEA	100%	1.3 (0.8–2.9)	LOQ 0.3	
	ENN B	100%	3.9 (1.7–8.8)	LOQ 1.3	
	ENN B1	67%	1.3 (< LOQ –1.5)	LOQ 1.0	
Ezekiel et al. (2022); Nigeria; n = 22 milk samples	BEA	100%	3.2 (1.0–12)	0.1/0.3	Multitoxin analysis conducted also in infant urines and in complementary food
	ENN B	72.7%	4.6 (0.7–10.1)	0.7/1.4	
	ENN B1	22.7%	0.5 (0.5–1.15)	0.5/1.0	
Braun et al. (2022); Nigeria; 23 mother-infant pairs; n = 75 breast milk samples	BEA	100% > LOD	3.6 ± 3.2 (0.9–18)	LOD 0.1	Mycotoxin intake estimates with breast milk are far lower than with plate-ready food
	ENN B	56% > LOD	5.1 ± 2.2 (0.7–12)	LOD 0.7	
	ENN B1	26% > LOD	0.6 ± 0.3 (0.5–1.5)	LOD 0.5	
Ayeni et al. (2024); Nigeria; n = 14 mother-infant pairs and n = 68 milk samples	BEA	87%	3/1 (0.1–67)	LOD 0.1	Multimycotoxin analysis
	ENN B	not reported	5/4 (0.7–13)	LOD 0.7	
	ENN B1		< LOQ	LOD 0.5	
	ENN A	not reported	2/2 (2–2)	LOD 0.5

**Table 4:** Analysis of BEA and ENNs in Human Tissue Samples

Authors/Country/ Sample origin	Analyte/Tissue	% positive (n of samples)	Mean ± SD (Range) ng/g	LOD/LOQ ng/g	Comments
Castell et al. (2024); Spain; n = 125 autopsy samples from 26 persons (age 29–83 years)	BEA			vary between the tissues, e.g. Liver: 0.002/0.006 Fat: 0.025/0.082	in-house validated method with dispersive liquid–liquid microextraction (DLLM) and HPLC-QqQ-MS/MS analysis
	Brain	15.4% (4/26)	0.1 ± 0.3 (ND–0.7)		
	Liver	4.0% (1/25)	not quantified (NQ)		
	Lung	50.0% (13/26)	0.4 ± 0.4 (ND–0.9)		
	Kidney	3.8% (1/26)	0.1 ± 0.3 (ND–1.5)		
	Heart	32.0% (8/25)	0.1 ± 0.3 (ND–1.3)		
	Fat	91.7% (22/24)	0.5 ± 1.1 (ND–3.9)		
Castell et al. (2024); Spain; n = 125 autopsy samples from 26 persons (age 29–83 years)	ENN B			vary between the tissues, e.g. Kidney: 0.002/0.006 Liver: 0.011/0.036	in-house validated method with DLLM and HPLC-QqQ-MS/MS analysis
	Brain	57.7% (15/26)	0.7 ± 0.6 (ND–1.2)		
	Liver	100% (25/25)	7 ± 13 (0.47–56.1)		
	Lung	92.3% (24/26)	0.8 ± 0.3 (ND–1.3)		
	Kidney	80.7% (21/26)	0.8 ± 0.4 (ND–1.7)		
	Heart	64.0% (16/25)	NQ (ND–NQ)		
	Fat	100% (24/24)	0.2 ± 0.8 (NQ–3.8)		
As above	ENN B1			vary between the tissues, e.g. Brain: 0.012/0.039 Heart: 0.021/0.069	as above
	Brain	46.2% (12/26)	0.2 ± 0.2 (ND–0.47)		
	Liver	88.0% (22/25)	1.3 ± 1.0 (ND–4.0)		
	Lung	80.8% (21/26)	0.5 ± 0.3 (ND–0.9)		
	Kidney	42.3% (11/26)	0.4 ± 0.5 (ND–1.5)		
	Heart	28.0% (7/25)	0.1 ± 0.3 (ND–1.3)		
	Fat	95.8% (23/24)	0.5 ± 1.1 (NQ–3.8)		
As above	ENN A			vary between the tissues, e.g. Lung: 0.029/0.096 Fat: 0.010/0.033	as above
	Brain	34.6% (9/26)	0.1 ± 0.1 (ND–0.30)		
	Liver	16.0% (4/25)	0.2 ± 0.4 (ND–1.0)		
	Lung	80.8% (21/26)	0.6 ± 0.3 (ND–1.0)		
	Kidney	23.1% (6/26)	0.2 ± 0.3 (ND–1.1)		
	Heart	24.0% (6/25)	0.03 ± 0.12 (ND–0.6)		
	Fat	83.3% (20/24)	4.7 ± 22.9 (ND–112.4)		
As above	ENN A1			vary between the tissues, e.g. Liver: 0.008/0.027 Kidney: 0.045/0.150	as above
	Brain	53.8% (14/26)	0.2 ± 0.2 (ND–0.46)		
	Liver	60.0% (15/25)	0.2 ± 0.4 (ND–1.0)		
	Lung	84.6% (22/26)	0.7 ± 0.6 (ND–1.8)		
	Kidney	42.3% (11/26)	0.3 ± 0.4 (ND–1.2)		
	Heart	32.0% (7/25)	NQ (ND–NQ)		
	Fat	95.8% (23/24)	1.0 ± 4.8 (ND–23.5)		

ND = not detected; NQ = not quantified

### Biomarkers/biomeasures of BEA occurrence in human samples

BEA was not measurable in **urine** samples collected from residents of European countries (Serrano et al. 2015; Escriva et al. 2017; Penczynski et al. 2022; Dasí-Navarro et al. 2024) or China (Liu et al. 2020) at the different detection levels (low µg/L or less) achieved by the analytical methods used (Table 1). Yet, BEA has been found in a high fraction (96%) of urines from Italy, in a range of 0.28–7.36 µg/L or 0.07–5.09 µg/g crea and a mean concentration of 0.70 ± 0.57 µg/L or 0.61 ± 0.49 µg/g crea (Mihalache et al. 2026).

Sensitive multi-mycotoxin methods have been also applied for BEA analysis in blood (Table 2). Yet, neither studies from Germany (Osteresch et al. 2017; Penczynski et al. 2022) found measurable BEA levels in serum (< 0.014 µg/L) nor a study of pregnant US women (Krausová et al. 2024), but it was detected in 5.9% of plasma samples in Bangladesh (Kyei et al. 2026). Moreover, recent studies from China reported BEA in >60% of plasma samples from adult males at mean level of 0.106 µg/L and 0.117 µg/L, respectively (Yang et al. 2024; Ning et al. 2024).

BEA was frequently detected in human milk samples from Austria (Braun et al. 2020a,b) and Nigeria (Braun et al. 2022; Ezekiel et al. 2022; Ayeni et al. 2024), albeit at very low concentrations, ranging from 0.1 to 67 ng/L and at similar median levels in both countries (Table 3). The data show that some of the BEA ingested by nursing mothers is apparently excreted with breast milk. Yet, the study authors note that mycotoxin exposure of exclusively breast-fed infants in Nigeria is likely lower than for those fed home-made complementary food (Braun et al. 2022; Ezekiel et al. 2022).

Recently, occurrence of BEA was analysed in n = 125 tissue samples from 26 persons undergoing forensic autopsies in Spain (Castell et al. 2024). The results indicate differences in its distribution in the human organism (Table 4), with the highest mean concentrations found in fat (0.5 ± 1.1 ng/g) and lung (0.4 ± 0.4 ng/g), lower in other organs (brain, kidney, heart), and not quantifiable in liver (< LOQ). The tissue distribution of BEA differs from that observed for ENN B and other ENNs (see below and Table 4).

### Biomarkers/biomeasures for occurrence of ENNs in human samples

The first analysis detected ENN B in 20/100 urine samples from German adults at low levels (Gerding et al. 2014), similar to a follow-up study in another German group, with mean concentrations of 0.012 ± 0.002 µg/L in 14% of urines (Gerding et al. 2015). With the same method, ENN B was found in 4% and 2% of samples from Haiti and Bangladesh (Table 1). Two method development reports are listed for completeness, yet analysis included only ten urine samples (Serrano et al. 2015; Escrivá et al. 2017). Further results from Europe indicate some differences in prevalence and analyte levels between countries and groups: Recent studies from Spain report an ENN B concentration range of 0.5–2.5 µg/L in 27% of n = 100 urines or 1.0–39.7 µg/g creatinine in 26% of n = 524 female urines (Dasí-Navarro et al. 2022 and 2024), and 0.07–4.45 µg/L in 68.75% of 80 urine samples from 20 volunteers (Gallardo-Ramos et al. 2024). More frequent detection of enniatins were recently reported in n = 300 urines from Italy, ranging between 0.65–7.14 µg/L for ENN B and 0.67–5.42 µg/L for ENN B1 and lower levels for ENN A and ENN A1 (Mihalache et al. 2026). Previous studies conducted in Sweden and Germany did not detect ENN B in urines of the respective study groups (Warensjö-Lemming et al. 2020; Penczynski et al. 2022), and very low levels (range ≥ 0.0001–0.014 µg/L) were reported for China (Liu et al. 2020). One may speculate that the above findings reflect differences in dietary ENN B intake levels between the study groups, although there is some uncertainty due to the use of spot or first morning void urines for multimycotoxin analysis by methods which also differed in sensitivity.

Rodriguez-Carrasco and colleagues have developed sensitive targeted methods for an analysis of ENN B and ENN B1 in urine which included also phase-I metabolites (termed M1–M12). Analysis of 300 urines from donors in Southern Italy showed a high prevalence for ENN B detection at an average concentration of 0.016 µg/L in a range of 0.006 to 0.391 µg/L (Rodriguez-Carrasco et al., 2018) and for ENN B1 a mean of 0.069 µg/L in a range of 0.007 to 0.429 µg/L (Rodríguez-Carrasco et al. 2020). By UHPLC-Q-Orbitrap HRMS analysis, also numerous metabolites of ENN B and ENN B1 were detected, and often at similar or higher urinary levels than the parent mycotoxin (Table 1). This indicates that biotransformation apparently contributes to the clearance of these mycotoxins and presence of their metabolites as suitable additional biomarkers of exposure.

Along with other mycotoxins, ENN B has been analyzed also in human blood. Studies from Europe found a high prevalence in serum samples obtained from German adults, Swedish adolescents and Portuguese workers (Table 2). Osteresch et al. (2017) who first established a sensitive method, reported a mean ENN B value of 0.033 µg/L (range 0.014–0.171 µg/L) in German adults, slightly lower than the mean level (0.048 µg/L) in a group of Portuguese workers (Viegas et al. 2018). ENN B was quantified in 84.1% of serum samples from Swedish adolescents, with a mean of 0.014 µg/L and max. level of 0.113 µg/L (Warensjö-Lemming et al. 2020). It was also frequently found in most (89 and 92%) samples from German vegans and omnivores (mean 0.017 and 0.024 µg/L, respectively), with no significant difference between these groups (Penczynski et al. 2022). In the latter two investigations, ENN B was below the LOD in urines of these study groups (Table 1) which suggests that blood is a better matrix for biomonitoring of ENNs. Of note, very small sample volumes obtained by finger pricking and dried blood/serum spots are now amenable for such an analysis (Osteresch et al. 2017; Warensjö-Lemming et al. 2020). Data on ENNs are also available from studies conducted outside of Europe (Table 2). Tesfamariam et al. (2022) report a range <LOD to 2.3 µg/L ENN B and a mean value <LOD (i.e. 0.57 µg/L) in serum samples of pregnant women in Ethiopia; yet details on data distribution are missing. A study from the United States on pregnant women measured ENN B in 97% of serum samples in a range of 0.01 to 0.08 µg/L (mean 0.02 µg/L), and about tenfold lower levels of ENN B1 in 12% of these samples (Krausova et al., 2024). In plasma samples from male adults in China, ENN B was present at mean levels of 0.003 (range 0.002–0.005) µg/L (Yang et al. 2024) and 0.006 (range 0.002–0.147) µg/L (Ning et al. 2024). These concentrations are lower than those reported in European studies. Recently, Kyei et al. (2026) reported frequent detection of ENN B in plasma samples from Bangladesh, with 91.7% above LOD (0.001 µg/L) and about one third above the LOQ.

Enniatins (ENN B, ENN B1) were found in the majority of breast milk samples from Austria and Nigeria at low to medium ng/L concentrations and in similar ranges as BEA (Table 3). An early analysis of breast milk from Spain detected ENNs (ENN A, ENNA1, ENN B and ENN B1) in 2 of 21 samples, with a reported range of 20 to 110 ng/mL (Rubert et al. 2014); the unit given in the report (ng/mL) is misleading and considered a reporting error (Warth et al., 2016). The first analysis of human milk from Nigeria detected ENN B in only 9% of the samples (Braun et al. 2018), whilst an optimized multimycotoxin method with higher sensitivity (Braun et al. 2020a) enabled more frequent detection of ENNs in follow-up studies. The improved method was applied to milk samples (n = 87) of one mother, collected after giving birth until day 211 postpartum, and samples (n = 15) of three Austrian mothers collected on 5 consecutive days (Braun et al. 2020b). The results in both groups showed similar ranges (1.3–8.6 and 1.7–8.8 ng/L) and mean (2.3 and 3.9 ng/L) values for ENN B; lower mean values were found for ENN B1 (1.9 and 1.3 ng/L, respectively) in these samples (Table 3). Analysis of milk samples from Nigeria (Ezekiel et al. 2022; Braun et al. 2022; Ayeni et al. 2024) found ENN B concentrations similar or slightly higher than those in Austria, whilst ENN B1 is detected less frequently and at lower levels. Exposure of breast-fed infants compared to those receiving infant formulae or home-made complementary food will be addressed in the Discussion section. 

The study from Spain on occurrence of BEA in n = 125 tissue samples from 26 persons undergoing forensic autopsies, reported also data for ENNs (Castell et al. 2024). Results for ENN B, the most frequently detected analyte, indicate a wide distribution in the human organism, with far higher levels found in liver (7±13 ng/g, range 0.47–56.1 ng/g tissue) than in other organs or in fat (values listed in Table 4). Data reported in the paper for ENN B1, ENN A and ENN A1 indicate less frequent occurrence and at lower levels in most tissues, except in fat (Castell et al. 2024). This investigation sheds some light on the distribution of these mycotoxins in human tissues.

Finally, occurrence of some mycotoxins has been investigated by means of HPLC-Q-TOF-HRMS in hair samples (n = 100) collected from hairdressing salons in Spain (Narváez et al. 2022). In this matrix, prevalence of detection ranged between 6 and 18% for the four ENNs, and 5% for BEA, with mean concentrations in positive detects between 16 and 46 ng/g hair. Yet, based on one pilot study only, it remains unclear whether such an approach in HBM may serve as long-term biomeasure of exposure to mycotoxins (Cramer et al. 2026).

## Discussion

### Biomonitoring data for BEA and ENNs in different matrices

Data collected over the last decade on presence of BEA in human samples (urine, blood, breast milk, tissues) are still scarce and vary with the matrix analyzed (Tables 1, 2, 3, 4). Not all multi-mycotoxin methods applied so far for urine and blood analysis have included BEA or could achieve the same sensitivity for detecting this analyte as for ENNs (Tables 1 and 2). Yet, a recent analysis detected BEA in a high percentage of Italian urines (n = 300) and at similar levels as the enniatins (Mihalache et al. 2026). Nonetheless, results available for BEA indicate that urine may not be the best matrix for biomonitoring which might be due to poor renal excretion of this mycotoxin in humans. This view is supported by a recent study where BEA and ENNs were present in 100% of pig feed samples, while observed at a far lower incidence in pig urines and only at low (ng/mg crea) levels (Castell et al. 2025). In rodent studies BEA was not detected in urine of mice treated *i.p.* on 3 days with 5 mg/kg bw, but present in serum and in tissues (Rodríguez-Carrasco et al. 2016). In urine of rats given 10 mg/kg bw BEA *per os* only a minute fraction (< 0.0005%) of the dose was excreted as parent compound within a day (Pandey et al. 2025a). On the other hand, some phase-I metabolites of BEA formed by liver microsomes were also found in rat urine samples, and based on peak area present at similar levels as BEA, up to 72 h after oral dosing at 10 mg/kg bw (Pandey et al. 2025a). Thus, the authors suggest metabolites as additional biomeasures of BEA exposure.

### BEA and ENNs in urine

Presence of ENNs has been reported in urine samples from various countries, with differences in concentration ranges for ENN B and ENN B1 between studies and less frequent detection of ENN A and ENN A1 (Table 1). Whether analyte patterns reflect dietary intake of these contaminants remains unclear also due to data gaps for human toxicokinetics of ENNs regarding their urinary excretion rates (Behr et al. 2025; Pandey et al. 2025b). Furthermore, an analysis of urines which included phase-I metabolites of ENN B and ENN B1 has detected numerous metabolites present at similar or higher levels than the parent compound (Rodríguez-Carrasco et al. 2018 and 2020). These in vivo findings concur with studies performed in vitro with human hepatic microsomes where monooxygenated, N-demethylated and dioxygenated/carboxylated forms of ENN B were characterized (Ivanova et al. 2011; Faeste et al. 2011), and metabolism of ENN B1 resulted in products of oxidative demethylation, hydroxylation, carbonylation and carboxylation (Ivanova et al. 2019). Whilst metabolites of ENNs are not yet available as reference compounds, it seems worth to include them in future biomonitoring studies.

### BEA and ENNs in blood

Analysis of blood (serum/plasma) found ENN B in most samples from Europe, Ethiopia, China and the US whereas BEA was only measured in two studies from China, and at higher concentrations than ENN B (Table 2). Differences in biomarker occurrence may be partly related to detection limits for both analytes, but also to food contamination in different countries with these mycotoxins. Studies in China report BEA as the predominant toxin in various cereals (corn, rice, wheat) and often present at higher levels than ENNs (Han et al. 2018; Lin et al. 2021), albeit the contamination pattern varies in different Chinese provinces (Ji et al. 2023). According to published data for European countries, BEA and ENNs are found in all grains investigated: In general, prevalences and concentrations are highest for ENN B and ENN B1, followed by ENN A, ENN A1 and BEA, and a similar picture of contamination was found for several cereal-based food products (Appendix A in EFSA 2014). A more recent analysis of wheat, durum wheat, triticale and barley observed ENN B as the most frequent contaminant (*68% of the total enniatin content*), followed by ENN B1 (*22%*), ENN A (*7%*) and ENN A1 (*3%*) present at variable levels and with the highest concentrations in all crops for the 2012 harvest in France (Orlando et al. 2019). Also, analysis of wheat harvested in 2020 and 2021 in several regions in Austria reveal the highest concentrations for ENN B and ENN B1, lower levels of ENN A1, ENN A and much less BEA (Reiter et al. 2024). These occurrence data in cereal crops are in line with biomarker data.

**Fig. 2:**
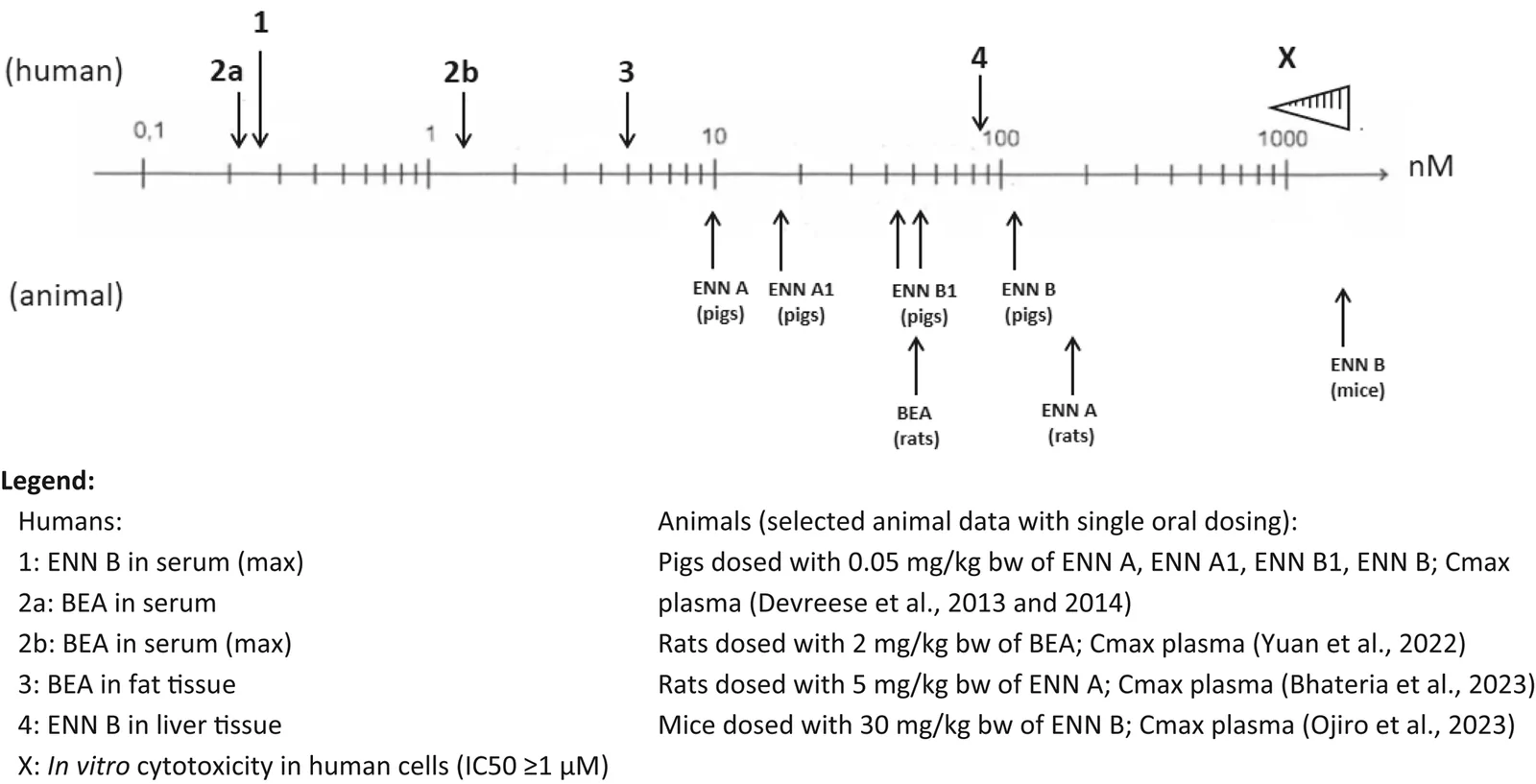
Concentrations present in human samples and levels of BEA and ENNs analyzed in some animal studies and those which elicit cytotoxicity in cells in vitro

The data obtained so far for blood provide first insights on circulating levels of mycotoxins, with average concentrations lower than 0.05 µg/L for ENN B in European samples, and 0.15 µg/L for BEA in Chinese samples (Table 2). The maximal values reported for ENN B (0.18 µg/L) and for BEA (1.12 µg/L) correspond^2^ to circulating concentrations of 0.28 nM and 1.43 nM, respectively. This may not reflect peak levels reached shortly after ingesting contaminated food, and metabolites detected in urine samples (see above) have not yet been analysed in plasma or serum. Nonetheless, these nM concentrations present in human blood (arrows 1 and 2a,b in Fig. 2) are many orders of magnitude lower than the µM mycotoxin concentrations (IC_50_ values of 1 µM or more) known to exert cytotoxicity in mammalian cells in vitro (data reviewed in Bertero et al. 2020; Behr et al. 2025).

Figure 2 also depicts HBM findings in blood (at unknown dietary intake levels) along with data reported for Cmax values in pigs and rodents after single oral dosing with BEA or ENNs. Considering the resemblance in gastrointestinal physiology between pigs and humans (Hatton et al. 2015), a comparison to the pig data for ENNs with levels between 10 and 115 nM after 0.05 mg/kg bw (Devreese et al. 2013, 2014) might indicate that human exposure is lower. Rodents dosed with 2 mg/kg bw BEA or 5 mg/kg bw ENN A or 30 mg/kg bw ENN B reached Cmax values in plasma of 53 nM, 170 nM or 1.6 µM, respectively (Yuan et al. 2022; Bhateria et al. 2022; Ojiro et al. 2023).

Figure 2 depicts human biomonitoring concentrations as well as values reported in some animal studies that differ in species, dosing conditions, sampling time and exposure duration, along with a nominal IC_50_ value for cytotoxicity. The illustration provides a descriptive context, but should not be misinterpreted to exclude chronic or noncytotoxic effects.

### BEA and ENNs in human milk

The group of Benedikt Warth pioneered the analysis of multiple mycotoxins in human milk and has investigated occurrence of BEA and ENNs in samples collected in Austria and Nigeria. According to data compiled in Table 3, emerging mycotoxins ingested by nursing mothers are partly excreted with breast milk, albeit at very low concentrations, ranging from 0.1 to 67 ng/L for BEA, 1.3 to 8.8 ng/L for ENN B and 0.5 to 1.9 ng/L for ENN B1. The levels present in human milk combined with milk consumption data for various age groups (EFSA 2017), allow to calculate infants exposure to these mycotoxins. For instance, using the maximal value for BEA of 67 ng/L, an average daily consumption of 0.8 L or a high consumption of 1.2 L milk for a 3-month-old infant with a body weight (bw) of 6.1 kg, would result in BEA exposures of 8.8 ng/kg bw/day or 13.2 ng/kg bw/day. But, considering mean BEA concentrations of 0.8 and 3.2 µg/kg found in complementary foods from Nigeria (Ojuri et al. 2019) or 2.1 µg/kg in cereal-based infant formulas from Portugal (de Sá et al. 2025), infants consuming such foods may be exposed to higher levels than breast-fed infants. Also, data on ENNs present in complementary infant foods (reviewed in Hernandez et al. 2021; de Sá et al. 2025) indicate human milk as best and safest source of nutrition.

### BEA and ENNs in human tissues

The first study on analysis of human tissues (Castell et al. 2024) provides some insights on internal mycotoxin concentrations resulting from continuous dietary exposure. BEA has been detected most frequently in fat with the highest value in the ng/g tissue range, followed by lung, whilst ENN B is the most prevalent mycotoxin in the body and found mainly in the liver (Table 4). Average and highest values reported for BEA in fat (0.5 and 3.9 ng/g or about ng/mL) or ENN B in liver (7 and 56.1 ng/g or about ng/mL) can be converted to nM concentrations of 0.63 and 4.9 nM for BEA or 11 and 88 nM for ENN B in these tissues, using ng/mL as proxy for ng/g tissue levels and the respective molecular weights. These mycotoxin concentrations found in human tissues (arrows 3 and 4 in Fig. 2) are far lower than low µM levels (triangle in Fig. 2) which exert cytotoxicity in vitro (data reviewed in Bertero et al. 2020; Behr et al. 2025). As stated before, Fig. 2 is just a descriptive comparison and should not be interpreted as evidence that current human exposure is safe.

So far metabolites of BEA and ENNs have not been analyzed in *human* tissues, yet may be present for instance in liver, the most active organ in biotransformation of xenobiotics. In liver and colon of mice treated *i.p.* on 2 days with 5 mg/kg bw ENN B, phase-I metabolites were found, with dioxygenated ENN B present at 3–10 fold higher levels than the parent molecule, but with no detection of metabolites in other tissues or biofluids analyzed at 24 h after the last dose (Rodríguez-Carrasco et al. 2016). As yet, there are no data on the toxicity of these metabolites in relation to their parent compound.

### Conclusions and recommendations

This first review on the presence of BEA and ENNs in biological matrices demonstrates internal exposure to these mycotoxins in several selected populations, with diet being the likely source. The data also provide some information on their fate in the human organism. Occurrence of BEA, ENNs and if possible analysis of their metabolites in different matrices is useful in planning further HBM studies and investigations on their toxicokinetics in humans. Further human toxicokinetic studies for BEA and for ENNs are needed for several reasons: (i) without such data, *e.g.* on urinary excretion rates, one cannot apply reverse dosimetry for calculating exposure based on urinary biomarker levels of parent compound and/or metabolites; (ii) plasma-concentration time curves after single dosing would help to define bioavailability and select suitable sampling intervals in future HBM studies. As controlled human dosing studies require permission by an ethics committee, also paired dietary-blood-urine studies or PBPK modelling should be considered. Since EFSA already noted missing toxicokinetic data as critical data gap in its previous opinion (EFSA CONTAM Panel 2014), several in vitro studies have provided new information: Incubations with hepatic microsomes or hepatocytes from various mammalian species, including humans, document efficient biotransformation of BEA (Yuan et al. 2022) and of ENNs (Bhateria et al. 2022; Faeste et al. 2011; Ivanova et al. 2011 and 2019). Thus, it would be useful to (iii) include these metabolites in future HBM studies to gain more insights on their presence and distribution in biosamples. Detection of phase-I metabolites in two HBM studies (Table 1) raises also the question (iv) whether biotransformation products of BEA and ENNs are similarly or less toxic than the parent mycotoxins?

Despite existing data gaps for in vivo toxicokinetics of BEA and ENNs in humans and lacking information on the toxicity of their metabolites, the present HBM data may already complement ongoing work to assess risks from exposure to these mycotoxins, *e.g.* for breast-fed infants. Moreover, internal mycotoxin levels measured in animal studies at known exposure levels can help to contextualize existing findings in human biosamples resulting from dietary intake. In this context, it is recommended to analyze biomarkers in blood and tissue samples from an ongoing project within PARC which assesses ENN B1 toxicity in rodents.

## Data Availability

No datasets were generated or analysed during the current study.

## Footnotes

1 The term *biomarker* is also often used for *biomeasures*, i.e. analytes for which a quantitative relationship between their levels in biological samples and intake has not yet been established (Cramer et al. 2026).

2 Converted to nM levels for ENN B (MW 639.4) at 180 ng/L or BEA (MW 784) at 1,120 ng/L.
